# Platelet function testing using the Multiplate analyzer after administration of aspirin in Aachen minipigs

**DOI:** 10.1371/journal.pone.0275756

**Published:** 2022-10-18

**Authors:** Christiane Franz, Lara Bender, Christoph Dorn, Thorsten Sichtermann, Jan Minkenberg, Maximilian Franko, Martin Wiesmann, Andrea Stockero, Omid Nikoubashman, Rebecca May, Hani Ridwan

**Affiliations:** Department of Diagnostic and Interventional Neuroradiology, University Hospital RWTH Aachen, Aachen, Germany; "INSERM", FRANCE

## Abstract

Knowledge of platelet function in pigs and the effectiveness of antiplatelet therapy is important to ensure proper transferability from animal studies to humans. Our aim was to (1) characterize baseline platelet function of Aachen minipigs using the bedside Multiplate analyzer, (2) compare baseline platelet function with Göttingen minipigs, and (3) characterize platelet inhibition within the first 5 minutes after intravenous administration of acetylsalicylic acid (ASA). We characterized the baseline platelet function and hematological parameters in 9 Aachen minipigs. Historical data of 8 unmedicated Göttingen minipigs were used for comparison of baseline values. Platelet inhibition in Aachen minipigs was tested 1–5 minutes after intravenous administration of 500 mg ASA. Multiplate examinations included the following tests: ASPI test (to assess the effect of ASA), adenosine-diphosphate-test (ADP test) and thrombin receptor activating peptide test (TRAP test). Median values and interquartile range (IQR) of the Multiplate baseline tests in Aachen minipigs were as follows: ASPI: 39 U (IQR = 21–71), ADP: 70 U (IQR = 48–73), and TRAP: 8 U (IQR = 6–9), whereas the values in Göttingen minipigs were as follows: ASPI: 70.5 U (IQR = 60–78), ADP: 51 U (IQR = 45–66), and TRAP: 6.5 U (IQR = 4–8). ASPI values of Göttingen minipigs were significantly higher than those of Aachen minipigs (p = 0.046). Intravenous administration of ASA in Aachen minipigs resulted in significant platelet inhibition after 1 minute, which remained stable over a period of 5 minutes (p≤0.038). Aachen minipigs appeared to have a high variance in arachidonic acid-mediated platelet aggregation. In Aachen minipigs, intravenous ASA administration resulted in immediate platelet inhibition.

## Introduction

In human medicine and research, antiplatelet drugs are common, for instance to prevent thrombotic or thromboembolic events after neurovascular or coronary interventions [1–3]. Likewise, platelet inhibition in pigs plays an important role, as they are used as animal models in various fields, such as neurointerventional, cardiovascular, trauma, and shock research and training [4–7].

In contrast to small animal species, pigs allow a good transferability from animal to human research due to their comparable body size, anatomy, and hemostatic and hemodynamic characteristics [5, 8, 9]. Aachen minipigs are a special breed, that is gaining more popularity in biomedical research because they represent a new, robust and non-specific pathogen free (non-SPF) pig race [10]. However, little is known about platelet function and the effect of antiplatelet drugs in Aachen minipigs. Since there are interspecies differences in platelet function and there is evidence for differences in hematological parameters between different pig breeds, previous results may not be transferable to Aachen minipigs [6, 7, 11–14].

Our aim was to characterize platelet function and the early effect of intravenous acetylsalicylic acid (ASA) on platelet function in Aachen Minipigs, using the Multiplate analyzer, one of the common bedside platelet aggregation tests that are used in human clinical routine and research. To further characterize platelet function in Aachen minipigs, we compared baseline values of unmedicated Aachen and Göttingen minipigs. To the best of our knowledge, no previous study has addressed the first 5 minutes of platelet inhibition after intravenous administration of ASA in pigs.

## Methods

### Experimental animals

All experiments were performed in accordance with the German Animal welfare law and the Directive 2010/63/EU on the Protection of Animals used for scientific Purposes. The experiments were carried out after receiving approval of the governmental animal care office (Landesamt für Natur, Umwelt und Verbraucherschutz Nordrhein-Westfalen, Recklinghausen, Germany) the corresponding approval number being AZ-81-02.04.2019.A412. Institutional guidelines for animal welfare and experimental conduct were followed. Animals were housed as previously described, except that they were fed twice daily with water ad libitum [7].

Both, the Aachen minipigs and the Göttingen minipigs were actually used for a medical device testing study, with the multiplate tests only being a sub-study. In summary, 17 minipigs were part of this study. We analyzed blood samples of 9 female miniature pigs (Aachen Minipigs, Gerd Heinrichs, Heinsberg-Karken, Germany; mean weight of 42.3kg ± 3.75 kg (mean ± SD); age of 17–21 months (range)) in the context of a medical device testing study with long-term follow-up (not yet published). These animals underwent two angiographies in total and were euthanized after the second angiography. A second group of 8 female miniature pigs (Ellegaard Göttingen Minipigs A/S, Dalmose, Denmark; mean weight of 38.1kg ± 12.6kg, mean age of 13 months ± 8.4 months) originated from a previous study [7]. This historical control group was included instead of conducting new experiments in terms of the three Rs of animal testing (Replacement, Reduction and Refinement) according to Russell & Burch [15]. From these animals, only the results of the Multiplate^®^ Analyzer before drug administration were used as additional baseline values for unmedicated pigs.

Only clinically healthy animals without premedication that could have interfered with our tests (antiplatelet or anticoagulant medication) were included in this study. Prior antibiotic treatment was no exclusion criterion. A severely reduced thrombocyte count (<100 x 10^9^/L) was an exclusion criterion that was not present in our cohort. Hence, no animals were excluded.

### Experimental design and procedures

Our study and the previous study by Heringer et al. (2019) were performed at the central institute for laboratory animal science of our hospital [7]. All experiments were performed under general anesthesia. Standard medication included premedication with azaperone (Stresnil 40 mg ad. us.vet.; Sanochemia Pharmazeutika AG, Neufeld, Austria), atropin (Atropinsulfat, B.Braun Melsungen AG, Melsungen, Germany), and ketamine (10% Ketavet ad. us. vet., Zoetis Deutschland GmbH, Berlin, Germany), followed by orotracheal intubation and placement of a urinary bladder catheter (non-surgical). During anesthesia, the animals were mechanically ventilated with an air-oxygen mixture. Anesthesia was maintained with isoflurane (1.5vol%) or propofol (Propofol 2% MCT Fresenius; Fresenius Kabi Deutschland GmbH, Bad Homburg, Germany) depending on the experimental setting. In all cases, fentanyl (Fentanyl-Janssen 0.5 mg, Janssen-Cilag GmbH, Neuss, Germany) was administered as an analgesic. During anesthesia, the animals were placed in the supine position and body temperature was maintained within physiological limits. Heart rate, ECG, and oxygen saturation (via pulse oximetry) were monitored throughout anesthesia. In chronic experiments, the recovery phase was closely monitored and postoperative analgesia carprofen was administered for a period of 24 hours (Rimadyl ad. us. vet., Zoetis Schweiz GmbH, Zürich, Switzerland). At the end of the experiments, euthanasia was performed by intravenous injection of sodium pentobarbital (Narcoren 16g/100ml; Merial GmbH, Hallbergmoos, Germany) during anesthesia. The Göttingen and Aachen minipigs were anesthetized and instrumented equally and the baseline samples were obtained at the same time point after instrumentation of the animals [7].

Arterial access was required as part of all experiments. For this purpose, the femoral artery was punctured without surgical exposure in the anesthetized pigs and an endovascular introducer sheath was inserted. Subsequently, blood sampling was performed via the sheath during anesthesia at the corresponding timepoints before proceeding with the endovascular procedure. Blood samples were obtained directly after placement of the sheath. As we performed the endovascular procedures after blood sampling, no resting period was required for the animals. Sample collection and analysis were performed under general anesthesia as described previously [7]. Multiplate analysis included the following tests: ASPI test (to assess the effect of ASA), adenosine-diphosphate-test (ADP test), thrombin receptor activating peptide test (TRAP test).

In the 9 Aachen minipigs, ASA was administered via the ear vein and the catheter was flushed with saline solution after the baseline sample. Platelet inhibition was induced via intravenous injection of 500 mg ASA (Aspirin^®^, Bayer, Leverkusen, Germany). Subsequent blood samples for Multiplate analysis were taken after 1, 2, 3, 4, and 5 minutes, respectively (i.e. t_1_, t_2_, t_3_, t_4_, t_5_).

For Multiplate analysis, 1.6 ml blood was filled into a hirudin-coated tube (Monovette-S, Sarstedt, Nümbrecht, Germany) and rested at room temperature. The fact that a single Multiplate analysis takes approximately 15 minutes while samples were taken every minute during the first five minutes resulted in a delay of sample processing (median = 104 minutes; IQR = 74–132 minutes). Platelet inhibition was measured by ASPI, ADP and TRAP test using the Multiplate Analyzer (Roche Diagnostics, Mannheim, Germany) according to the manufacturer’s instructions. Briefly, as previously described in Heringer et al. (2019) [7], 300 μl of hirudin blood was first diluted with prewarmed (37°C) isotonic sodium chloride. After an incubation period of 3 minutes at 37°C, 20 xl of the corresponding agonist (arachidonic acid (AA), adenosine diphosphate (ADP), or thrombin receptor-activating peptide 6 (TRAP-6)) was added to each. Only commercially available standard reagents were used (Roche Diagnostics, Mannheim, Germany). The actual measurement time for each test was 6 minutes. The detected aggregation was expressed as aggregation units (AU), aggregation velocity (AU/min) and area under the curve (AUC, AU*min [U]). For further analysis, the area under the curve was chosen as output parameter, which is quantified in arbitrary aggregation units AUC [U].

Blood sampling for hematological examination was performed with EDTA-coated tubes (Sarstedt, Nümbrecht, Germany) and carried out using a Celltac α MEK-6550K system (Nihon Kohden Europe, Rosbach, Germany) at the central institute of laboratory animal science of our institution. Measured parameters were as follows: White blood cell count, red blood cell count, platelet count, hemoglobin, and hematocrit.

Since platelet concentration may affect Multiplate results, we correlated baseline hematological and platelet function values. Because it is highly unlikely that blood sampling and aspirin medication in our experiment affected hematological results, we did not perform further correlations.

### Statistical analysis

Shapiro-Wilk tests showed that multiplate results were not consistently normally distributed. Therefore, multiplate results are indicated as median and interquartile range (IQR) as well as mean and standard deviation (SD) and data distribution is indicated in the respective table. Platelet counts were not normally distributed and were indicated as median and IQR. We performed Mann Whitney U tests for the comparison of Aachen and Göttingen minipigs and paired Wilcoxon signed-rank tests for comparisons of different time points after ASA administration (i.e. t_0_ vs t_1_, t_0_ vs t_2_, t_0_ vs t_3_, t_0_ vs t_4_, t_0_ vs t_5_, t_1_ vs t_2_, t_2_ vs t_3_, t_3_ vs t_4_, t_4_ vs t_5_, for ASPI, ADP and TRAP, respectively). We considered a correction for multiple testing but because we did not compare all time points at the same time, but rather compared single time points to each other, a correction for multiple testing was not deemed necessary in our case [16, 17]. Outliers were not excluded. Spearman’s correlation coefficient was used for correlation between thrombocyte count and ASPI baseline values. All tests were two-tailed. P-values under the α-level of 0.05 were defined as significant. SPSS 27 software (IBM, San Jose, California, USA) was used for statistical analyses and diagrams.

## Results

### Hematology

The Aachen minipigs (n = 9) in our study had platelet counts with a median of 296 x 10^9^/L (range = 196 x 10^9^/L -338 x 10^9^/L and IQR = 290–329 x 10^9^/L) and in eight out of nine pigs, the platelet counts were within the reference range of the investigating veterinary laboratory (220–620 x 10^9^/L). In one case, the platelet count was slightly below the reference value at 196 x 10^9^/L. This case was included in our analysis. Full results are presented in Table 1.

**Table 1:** Hematology results and Multiplate baseline values of Aachen minipigs (AMP) at t_0_.

Minipig no.	1	2	3	4	5	6	7	8	9	Ref.^a^
Leukocytes (x10^3^/μl)	10.0	8.0	8.4	4.4	9.4	8.7	10.3	10.4	6.5	10.0–22.0
Erythrocytes (x10^6^/μl)	4.70	3.72	2.77	3.12	3.54	4.76	4.96	4.07	4.48	5.0–8.0
Thrombocytes (x10^3^/μl)	329	301	296	297	196	290	329	289	338	220–620
Hemoglobin (g/dL)	10.1	8.0	6.5	7.4	7.8	10.3	10.1	8.7	8.9	10.0–16.0
Hematocrit (%)	29.6	23.7	19.7	22.5	23.0	30.6	29.6	25.2	26.2	33–45
Multiplate ASPI	43	15	39	9	27	37	75	85	66	-
Multiplate ADP	28	85	27	67	70	73	68	71	73	-
Multiplate TRAP	4	9	8	1	9	10	9	8	8	-

^a^. Reference values from the veterinary laboratory at the central institute for laboratory animals of our hospital.

### Reference values of unmedicated pigs

Median baseline values of the Multiplate subtests in Aachen minipigs were as follows: ASPI = 39 U (IQR = 21–71), ADP = 70 U (IQR = 48–73) and TRAP = 8 U (IQR = 6–9). Full results and corresponding reference values of Göttingen minipigs are presented in Table 2. A between unmedicated Aachen and Göttingen minipigs significantly lower ASPI values in Aachen (p = 0.046). For this reason, the two groups were not pooled as common baseline values. The comparison of the baseline values for ADP and TRAP did not yield a significant difference (p = 0.139 and p = 0.370, respectively). The corresponding statistical parameters can be found in Table 2.

**Table 2:** Baseline Multiplate readings for unmedicated Aachen and Göttingen minipigs.

	ASPI		ADP		TRAP	
	Aachen minipigs^a^	Göttingen minipigs^a^	Aachen minipigs^b^	Göttingen minipigs^a^	Aachen minipigs^b^	Göttingen minipigs^a^
Median (IQR)	39 (21–71)	70.5 (60–78)	70 (48–73)	51 (45–66)	8 (6–9)	6.5 (4–8)
mean (±SD)	44.0 (±26.35)	69.4 (±12.39)	62.4 (±-20.48)	52.6 (±16.3)	7.3 (±2.92)	6.8 (±3.15)
range	9–85	49–88	27–85	22–75	1–10	4–13
p	0.046		0.139		0.370	

Multiplate results in Aachen minipigs and Göttingen minipigs at t_0_.

^a^: normal distribution;

^b^: no normal distribution.

Because data were not consistently normally distributed, we applied the Mann Whitney U test for comparisons between Aachen and Göttingen minipigs.

Spearman correlation between thrombocyte count and ASPI baseline values was positive (0.395) but not significant (p = 0.293).

### Intravenous application of ASA

#### ASPI test

**Fig 1:**
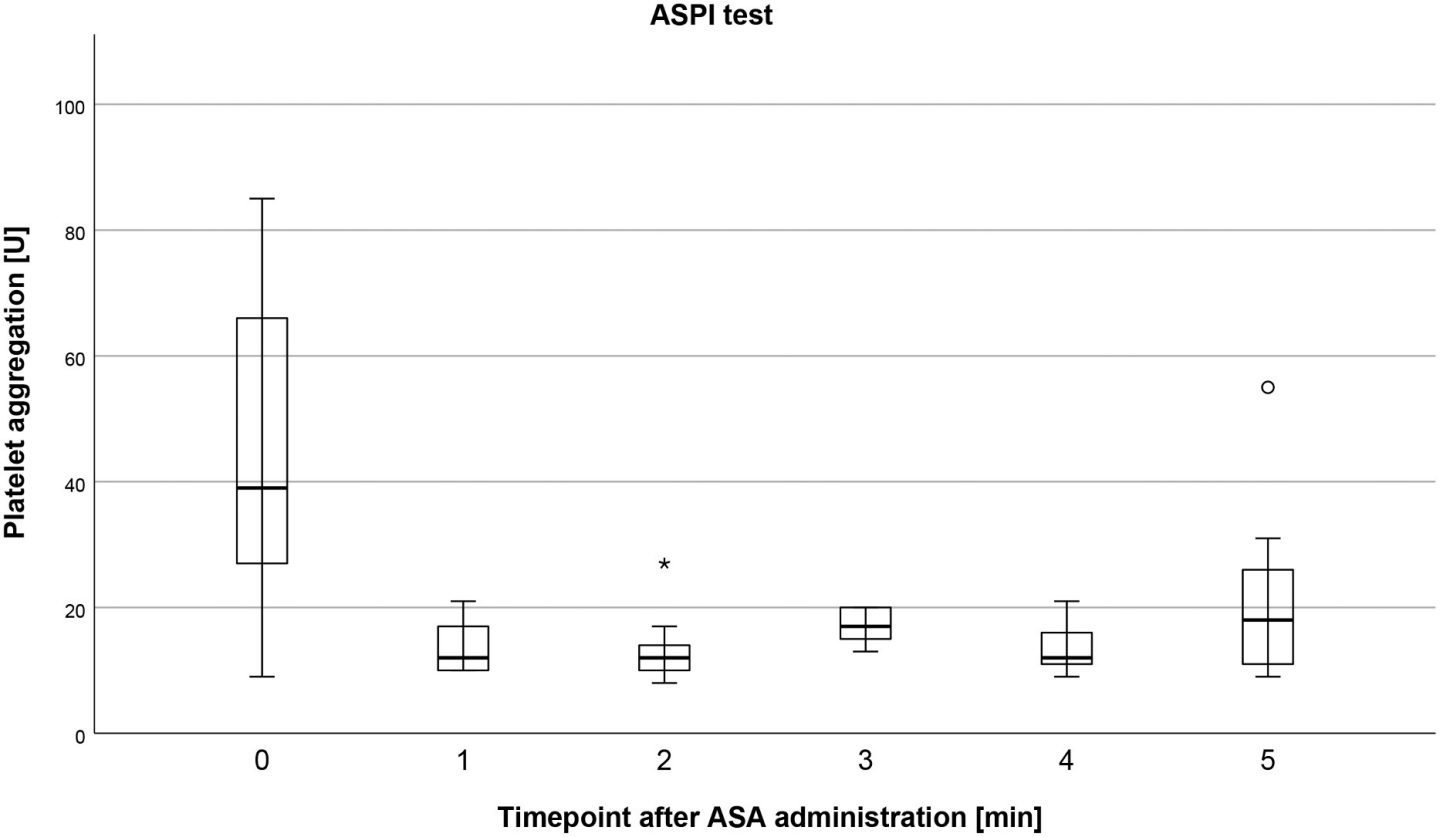
Boxplot depicting the effects of acetylsalicylic acid (ASA) on platelet function over five minutes measured by the Multiplate ASPI test. One minute after intravenous administration of 500 mg acetylsalicylic acid (ASA) platelet function of minipigs was significantly reduced in the ASPI test (which assess the effect of acetylsalicylic acid, ASA). Results are represented in arbitrary aggregation units AUC [U]. Differences between one and five minutes after administration of ASA were not significant (p>0.05). Circles represent outliers (more than 1.5 times the interquartile range) and asterisks represent extreme outliers (more than 3 times the interquartile range).

ASPI test at 1 minute after administration of ASA yielded a median of 12 U (range = 10–21, IQR = 10–18, mean = 13.67, SD = 4.09). ASPI test at 2 minutes after administration of ASA yielded a median of 12 U (range = 8–27, IQR = 10–16, mean = 13.44, SD = 5.77). ASPI test at 3 minutes after administration of ASA yielded a median of 17 U (range = 13–20, IQR = 15–20, mean = 17.11, SD = 2.57). ASPI test at 4 minutes after administration of ASA yielded a median of 12 U (range = 9–21, IQR = 11–16, mean = 13.56, SD = 3.64). ASPI test at 5 minutes after administration of ASA yielded a median of 18 U (range = 9–55, IQR = 11–29, mean = 22.00, SD = 14.41). (Fig 1).

#### ADP test

**Fig 2:**
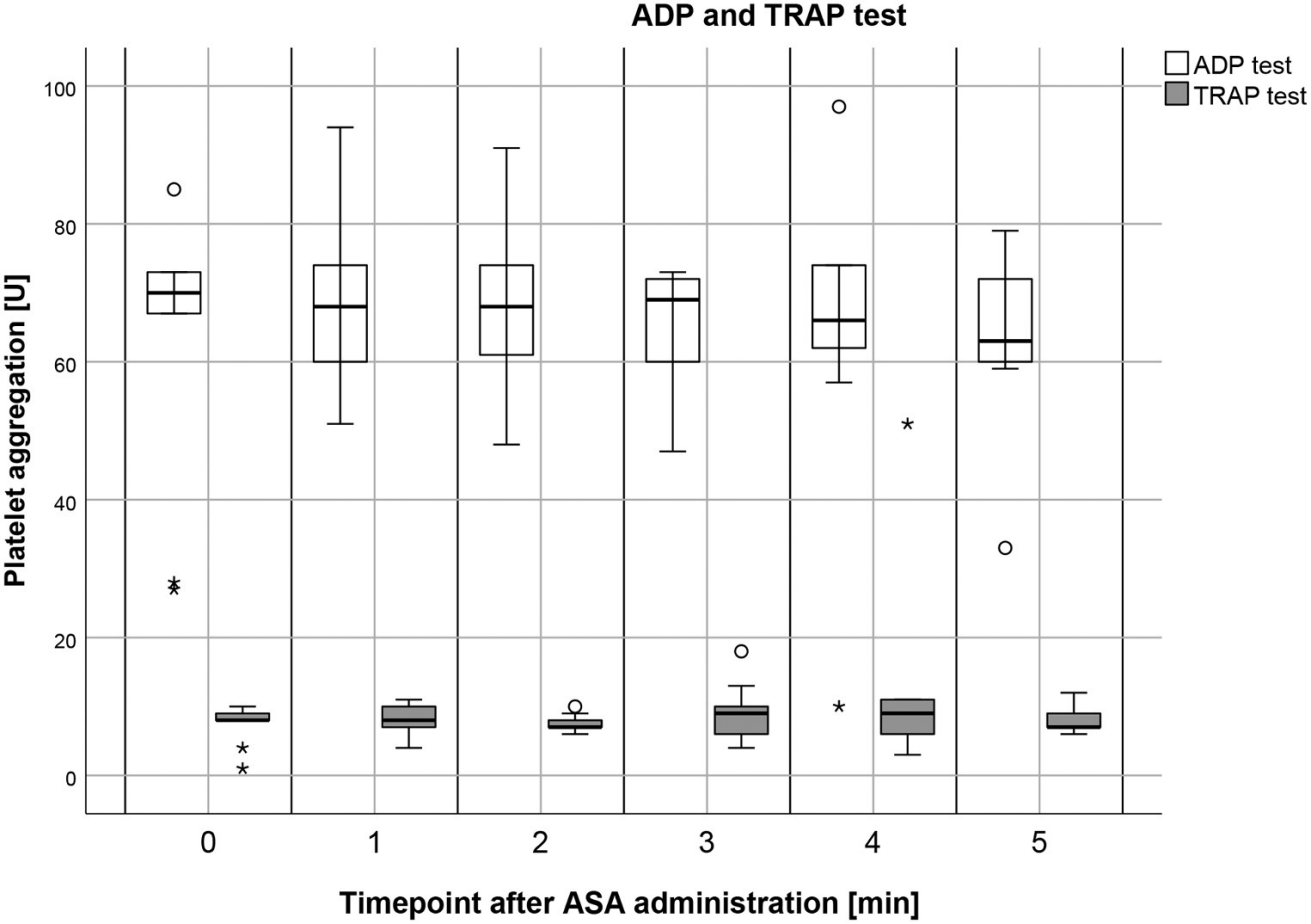
Boxplot depicting the effects of acetylsalicylic acid (ASA) on platelet function over five minutes measured by the Multiplate ADP and TRAP test. Results are represented in arbitrary aggregation units AUC [U]. Intravenous administration of 500 mg ASA did not reveal statistical differences between the different timepoints of ADP tests (adenosine-diphosphate) and TRAP tests (thrombin receptor activating peptide) (p≥0.213). Circles represent outliers (more than 1.5 times the interquartile range) and asterisks represent extreme outliers (more than 3 times the interquartile range).

ADP test at 1 minute after administration of ASA yielded a median of 68 U (range = 51–94, IQR = 57–77, mean = 68.11, SD = 13.48). ADP test at 2 minutes after administration of ASA yielded a median of 68 U (range = 48–91, IQR = 57–77, mean = 67.44, SD = 13.32). ADP test at 3 minutes after administration of ASA yielded a median of 69 U (range = 47–73, IQR = 54–73, mean = 64.33, SD = 10.46). ADP test at 4 minutes after administration of ASA yielded a median of 66 U (range = 10–97, IQR = 60–74, mean = 63.78, SD = 23.22). ADP test at 5 minutes after administration of ASA yielded a median of 63 U (range = 33–79, IQR = 60–72, mean = 62.89, SD = 13.05). (Fig 2).

#### TRAP test

TRAP test at 1 minute after administration of ASA yielded a median of 8 U (range = 4–11, IQR = 7–10, mean = 8.22, SD = 2.11). TRAP test at 2 minutes after administration of ASA yielded a median of 7 U (range = 6–10, IQR = 7–9, mean = 7.56, SD = 1.33). TRAP test at 3 minutes after administration of ASA yielded a median of 9 U (range = 4–18, IQR = 5–12, mean = 9.00, SD = 4.441.48). TRAP test at 4 minutes after administration of ASA yielded a median of 9 U (range = 3–51, IQR = 6–11, mean = 12.67, SD = 14.64). TRAP test at 5 minutes after administration of ASA yielded a median of 7 U (range = 6–12, IQR = 7–10, mean = 8.11, SD = 1.90). (Fig 2).

### Comparisons of multiplate results at different time points

Intravenous application of ASA (Aachen Minipigs, n = 9) resulted in a significant decrease of the ASPI test values with a median of 39 U in unmedicated pigs (t_0_) and with median values of 12 U, 12 U, 17 U, 12 U, and 18 U at all other time points (vs t_1_, t_2_, t_3_, t_4_, t_5_), respectively (p≤0.038). The other comparisons (t_1_ vs t_2_, t_2_ vs t_3_, t_3_ vs t_4_ and t_4_ vs t_5_), did not yield significant differences between these time points (p≥0.057). Intravenous application of ASA (Aachen Minipigs, n = 9) did not yield significant differences in the ADP test values between the different timepoints (t_0_ vs t_1_, t_0_ vs t_2_, t_0_ vs t_3_, t_0_ vs t_4_, t_0_ vs t_5_, t_1_ vs t_2_, t_2_ vs t_3_, t_3_ vs t_4_ and t_4_ vs t_5_) (p≥0.213). The median values of ADP tests were as follows: t_0_ = 70 U, t_1_ = 68 U, t_2_ = 68 U, t_3_ = 69 U, t_4_ = 66 U, and t_5_ = 63 U.

Intravenous application of ASA (Aachen minipigs, n = 9) did not yield significant differences in the TRAP test values between the different timepoints (t_0_ vs t_1_, t_0_ vs t_2_, t_0_ vs t_3_, t_0_ vs t_4_, t_0_ vs t_5_, t_1_ vs t_2_, t_2_ vs t_3_, t_3_ vs t_4_ and t_4_ vs t_5_) (p≥0.342). The median values of TRAP tests were as follows: t_0_ = 8 U, t_1_ = 8 U, t_2_ = 7 U, t_3_ = 9 U, t_4_ = 9 U, and t_5_ = 7 U.

## Discussion

Knowledge of platelet function and the efficacy of antiplatelet therapy is important to ensure proper transferability from animal studies to humans. We have characterized the platelet function in pigs using the Multiplate analyzer and have shown that this can be used to detect reduced platelet function after intravenous ASA administration.

The mean baseline value of the ASPI test is significantly lower in Aachen minipigs than in Göttingen minipigs (44.00 ± 26.35 U and 69.38 ± 12.39 U, respectively, p = 0.046). Looking at the distribution of the ASPI test results in Aachen minipigs, our results suggest that the baseline values in Aachen minipigs have a larger variance than in Göttingen minipigs. Even though our sample size with 9 minipigs is possibly too small to draw definite conclusions, the results imply a higher variability in arachidonic acid-mediated platelet aggregation in Aachen minipigs. In fact, there were very low and very high ASPI values, ranging from 9 to 85 U. In two cases, low initial values (9 and 15 U) remained low even after administration of ASA. This may indicate a reduced arachidonic acid-mediated platelet function in some Aachen minipigs. Interestingly, these two minipigs had rather low thrombocyte counts, but correlation between thrombocyte count and ASPI baseline values was positive but not significant (p = 0.293). As various authors report that extremely low platelet counts (<100 x 10^9^/L platelet range) can lead to reduced values of Multiplate test results, the correlation between decreased platelets and deviating Multiplate results in Aachen minipigs may be a potential target for future studies [6, 18, 19].

It is noteworthy that administration of ASA reduced ASPI test values in all pigs, except in the two minipigs that had already low baseline values. In these, the ASPI values remained low after intravenous administration of ASA. Intravenous administration of ASA reduced even high baseline values in the ASPI test of Aachen minipigs below human therapeutic limits of 30 U [20]. With regards to the dynamics of platelet inhibition with ASA, statistically significant differences were found between the baseline (t_0_) and all following time points (t_1,_ t_2,_ t_3,_ t_4,_ t_5_) after treatment. This indicates that platelet inhibition is already fully effective after one minute (p≤0.038) and remains effective afterwards. The median baseline value of 39 U was reduced to values between 12 and 18 U, which are all below human therapeutic limits of 30 U. These results are in line with our expectations, as it is known that aspirin is effective 30 seconds after intravenous administration in humans [21]. In pigs, however, systematic data are lacking: Johnson et al. (1995) reported a peak serum concentration of ASA derivates 15 minutes after intravenous administration of 300 mg in six Yorkshire pigs. The authors did not perform any earlier measurements. Their study does thus not allow any conclusions to be drawn about the effect of ASA within the first 15 minutes [22]. Another study dealing with dual antiplatelet therapy with ASA and ticagrelor in Swedish landrace pigs investigated platelet aggregation over time with a Multiplate analyzer without analyzing the effects immediately after ASA administration [23]. Hence, our results that show that intravenous administration of ASA is immediately effective, provide evidence that the effects of intravenous ASA administration are comparable to humans [21].

To our surprise, in contrast to previous results there was no significant decrease in ADP values after administration of ASA. Heringer et al. (2019) conducted a study with German Landrace and Göttingen minipigs (n = 12) and reported a significant decrease in ADP values after ASA administration as an unexpected result [7]. These differences may either be coincidental or may indicate different mechanisms of platelet aggregation in Aachen and Göttingen minipigs, respectively. Although there is also evidence in humans for a possible effect of ASA on the ADP test, our results must be interpreted with caution given the small sample sizes in both studies [24].

As expected, the TRAP test yielded low values, as TRAP was not expected to induce platelet aggregation Aachen minipigs. While arachidonic acid (ASPI test) and ADP (ADP test) have been shown to work reliably in pigs, the thrombin-receptor activator peptide (TRAP) is a specific peptide derived from human thrombin [7]. We used TRAP-6, which has been shown to be unable to induce platelet aggregation in pigs [25]. Several studies have been conducted on the effect of different TRAP subtypes on several species, but the exact mechanism remains unclear and in general there seem to be large species differences [7, 13, 25, 26].

In conclusion, our results indicate that platelet function in Aachen minipigs can be assessed with the Multiplate^®^ analyzer (ASPI test and ADP test) and that Aachen minipigs appear to have a high variance in arachidonic-acid-mediated platelet aggregation. Lower baseline ASPI test values in Aachen minipigs implies that the baseline arachidonic-acid-mediated platelet function of Aachen minipigs may be lower than in Göttingen minipigs. In Aachen minipigs, intravenous ASA administration resulted in immediate platelet inhibition which remained stable during an observation period of five minutes.

### Limitations

A limitation of our study is the small sample size, which is owed to the restrictive use of animals according to the three Rs (Replacement, Reduction and Refinement) of Russell & Burch.

Another potential limitation is the relatively short time frame, which was the focus of our study. As it is known that ASA inhibits platelet aggregation irreversibly and because we performed subsequent endovascular procedures that could have interfered with our results (including administration of heparin and use of heparinized saline during intervention) we focused on early effects of ASA and refrained from taking blood samples at the end of our experiments [27].

The varying storage times are a possible confounder of our results, as some authors have reported varying storage times to affect platelet function assays [28, 29]. However, we feel confident that this only had a minor impact on our results, because there are several other studies with no significant differences when resting periods reach up to 12 hours [27, 30, 31].

Even though our results must be interpreted with caution, we consider our results relevant and the limitations acceptable given the increasing demand for minipigs in biomedical research.

## Supporting information

S1 File Listed are all the results of the Multiplate analyzer measurements (ASPI, ADP, TRAP) in Aachen minipigs at different timepoints. (PDF)
