# Supplementary material for: Platelet function testing using the Multiplate analyzer after administration of aspirin in Aachen minipigs
Source: PLoS One. 2022 Oct 18;17(10):e0275756. doi: 10.1371/journal.pone.0275756 (PMC9578582; doi:10.1371/journal.pone.0275756)
Supplement: S1 File — (PDF) [file pone.0275756.s001.pdf]

Unmedicated Aachen minipigs			
t_0			
ID	ASPI	ADP	TRAP
	AUC [U]	AUC [U]	AUC [U]
1	43	28	4
2	15	85	9
3	39	27	8
4	9	67	1
5	27	70	9
6	37	73	10
7	75	68	9
8	85	71	8
9	66	73	8

Aachen minipigs after receiving ASA (500 mg iv)			
t_1			
ID	ASPI	ADP	TRAP
	AUC [U]	AUC [U]	AUC [U]
1	14	94	4
2	21	80	8
3	10	53	10
4	11	68	9
5	17	51	11
6	18	64	10
7	10	60	7
8	10	74	8
9	12	69	7

Aachen minipigs after receiving ASA (500 mg iv)			
t_2			
ID	ASPI	ADP	TRAP
	AUC [U]	AUC [U]	AUC [U]
1	27	74	9
2	10	80	8
3	8	61	6
4	13	91	7
5	17	53	8
6	11	48	6
7	12	70	7
8	9	62	7
9	14	68	10

Aachen minipigs after receiving ASA (500 mg iv)			
t_3			
ID	ASPI	ADP	TRAP
	AUC [U]	AUC [U]	AUC [U]
1	20	73	18
2	18	72	9
3	15	47	8
4	20	60	6
5	20	65	13
6	17	48	4
7	16	73	10
8	13	72	4
9	15	69	9

Aachen minipigs after receiving ASA (500 mg iv)			
t_4			
ID	ASPI	ADP	TRAP
	AUC [U]	AUC [U]	AUC [U]
1	9	97	9
2	14	74	10
3	12	10	51
4	11	71	5
5	16	62	11
6	21	57	8
7	11	63	6
8	12	66	3
9	16	74	11

Aachen minipigs after receiving ASA (500 mg iv)			
t_5			
ID	ASPI	ADP	TRAP
	AUC [U]	AUC [U]	AUC [U]
1	31	63	7
2	11	72	9
3	9	33	7
4	26	59	6
5	17	61	10
6	21	60	7
7	55	67	8
8	10	72	7
9	18	79	12
